# The mechanism of linear ubiquitination in regulating cell death and correlative diseases

**DOI:** 10.1038/s41419-023-06183-3

**Published:** 2023-10-10

**Authors:** Liyuan Gao, Wei Zhang, Xiao hui Shi, Xiaoyan Chang, Yi Han, Chundi Liu, Zhitao Jiang, Xiang Yang

**Affiliations:** Zhangjiagang TCM Hospital Affiliated to Nanjing University of Chinese Medicine, Suzhou, China

**Keywords:** Cell death, Cell signalling

## Abstract

Linear ubiquitination is a specific post-translational modification in which ubiquitin is linked through M1 residue to form multiple types of polyubiquitin chains on substrates in order to regulate cellular processes. LUBAC comprised by HOIP, HOIL-1L, and SHARPIN as a sole E3 ligase catalyzes the generation of linear ubiquitin chains, and it is simultaneously adjusted by deubiquitinases such as OTULIN and CYLD. Several studies have shown that gene mutation of linear ubiquitination in mice accompanied by different modalities of cell death would develop relative diseases. Cell death is a fundamental physiological process and responsible for embryonic development, organ maintenance, and immunity response. Therefore, it is worth speculating that linear ubiquitin mediated signaling pathway would participate in different diseases. The relative literature search was done from core collection of electronic databases such as Web of Science, PubMed, and Google Scholar using keywords about main regulators of linear ubiquitination pathway. Here, we summarize the regulatory mechanism of linear ubiquitination on cellular signaling pathway in cells with apoptosis, necroptosis, autophagy, pyroptosis, and ferroptosis. Intervening generation of linear ubiquitin chains in relative signaling pathway to regulate cell death might provide novel therapeutic insights for various human diseases.

## Facts

- LUBAC as the sole E3 ubiquitin ligase of linear ubiquitination can regulate NF-κB signaling and other regulation pathways.
- Linear ubiquitin chain mediated modification of protein is involved in regulating target cell autophagy, apoptosis, pyroptosis, necroptosis, and ferroptosis.
- Interventions for different diseases targeting linear ubiquitin chain modification have been carried out in recent years.

## Open Questions

- What is linear ubiquitination, and how it works?
- How does LUBAC participate in NF-κB signaling and other regulation pathways?
- Are there unknown mechanisms and signaling pathways about linear ubiquitination regulating various cell death?
- What is the relationship between linear ubiquitin modification and several diseases?

## Introduction

**Fig. 1:**
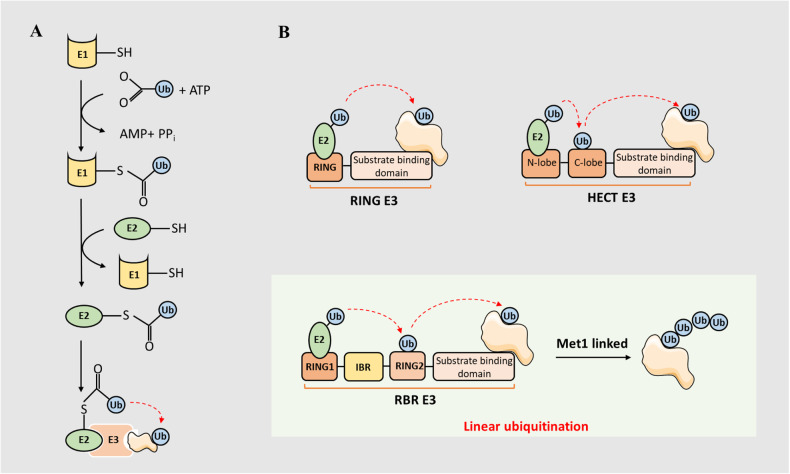
The canonical ubiquitination cascade reaction and linear polyubiquitin chain formation. **A** The E1 enzyme combines to ubiquitin with a thioester bond and consume an ATP. Then the E2 ubiquitin-conjugating enzyme catalyzes the transfer of ubiquitin from E1-thio-Ub to E2 via a transesterification reaction. Finally, ubiquitin on E2-thio-Ub can be transferred to substrate under E3 ligases catalysis. **B** The E3 ligases catalysis can be divided into three kinds: (1) The RING family E3 ligases can directly transfer ubiquitin from E2-thio-Ub to substrate. (2) The HECT family E3 ligases are able to conjugate the ubiquitin with their active domain firstly, and then deliver them to adjacent substrate. (3) The RBR family E3 ligases hold specific RING1-IBR-RING2 domain, in which the RNG1 domain can recognize E2-Ub for transferring ubiquitin from E2 enzyme to RING2 domain and then the ubiquitin is further transferred to substrate. The linear ubiquitination is catalyzed by RBR family E3 ligase LUBAC companied by Met 1 linked polyubiquitin chains.

Ubiquitin, a conserved 76-amino acid protein, is widely distributed in eukaryotic cells, and is covalently attached to other proteins which completing its function of misfolding for degradation or modifying for stabilization. Modification of proteins with ubiquitin requires three enzymes in cascade reactions, involving the activity of E1 ubiquitin-activating enzymes, E2 ubiquitin-conjugating enzymes, and E3 ubiquitin ligases [1]. Firstly, the cysteine sulfhydryl group of E1 enzyme is bound to ubiquitin glycine carboxyl group via the formation of a thioester bond accompanied by an ATP expenditure. Then, the E2 enzyme catalyzes the transfer of ubiquitin from E1-thio-Ub to cysteine active site of the E2 via a transesterification reaction. Next, ubiquitin on E2-thio-Ub conjugation can be delivered to protein substrate in two mechanisms depending on three species of the E3 ligase. For E3 ligases in the RING family, ubiquitin is directly transferred from E2-thio-Ub to the substrate. For E3 ligases in the HECT family, ubiquitin is delivered to the cysteine active site of E3 ligase at C-lobe domain before being transferred to substrate [2]. The E3 ligases in the RBR family contain RING1-IBR-RING2 domain. The RING1 domain can recognize E2-Ub and catalyze the transfer of ubiquitin from E2 enzyme to RING2 domain via the formation of thioester bond like the HECT family, then ubiquitin continues to translocate to the substrate [2]. The canonical ubiquitination cascade reaction and linear polyubiquitin chain formation is illustrated in Fig. 1. Some researchers also believe that the E4 ligase exists in ubiquitination. The E4 ligase is either complementary to E3 ligases, especially the RING E3 ligases, by prolonging polyubiquitin chain, or simulating the function of E3 ligases under certain circumstances [3, 4].

The human genome is estimated to encode 2 E1 enzymes, about 38 E2 enzymes and more than 600 E3 ligases [2]. Protein ubiquitination can be divided into several depending on the mode and amount of ubiquitin linked to substrate. Modification of a target protein may act as a single ubiquitin on single lysine of substrate (monoubiquitination), a single ubiquitin on multiple lysine (multiubiquitination), or as homotypic even heterotypic chains in which the ubiquitin initially linked to lysine of substrate proteins is coupled to another ubiquitin via an isopeptide bond formed between the C-terminal carboxyl group of glycine residue and the N-terminal ε-amino group of lysine residue (polyubiquitination) [5]. Ubiquitin has seven lysine residues (K6, K11, K27, K29, K33, K48, and K63) for polyubiquitin chain linkage. The types of poly-Ub chains are named by the lysine residue from ubiquitin such as Lys48-linked chains. In addition, linear polyubiquitination is different from others, in which ubiquitin is coupled via its N-terminal methionine (M1) residue and to another ubiquitin glycine, and it’s also called M1-linked chain polyubiquitination. In particular, the linear ubiquitin chain is generated by the sole E3 ubiquitin ligase LUBAC. Ubiquitination is a reversible post-transcriptional modification process, and it is reversed by DUBs, which remove ubiquitin from substrate. Approximately 100 different DUBs have been identified so far [6], but only OTULIN and cylindromatosis (CYLD) can remove M1-linked ubiquitin chain from substrate.

Different ubiquitin chain topologies adopt unique compact governed by the type of modified sites as well as the length of ubiquitin chain what has been summarized as “ubiquitin code” [7, 8]. Besides, ubiquitin on substrate can also endure small-molecule post-translational modifications such as phosphorylation [9, 10], acetylation [11–13], and ribosylation [14–16] which contribute to the increasing complexity of ubiquitin code. The metabolic stability or non-proteolytic function can be altered once the ubiquitin attached to substrate. The type of polyubiquitin chain determines the fate of the substrate protein. Recent studies suggest that K11-and K48-linked chains guide the substrate for proteasomal degradation, while K63- and M1-linked chain act as scaffolds for modifying proteins. The functions of other four ubiquitin linkage types (K6, K27, K29, and K33) are being revealed gradually [7]. Among them, M1-linked ubiquitin chain (linear ubiquitin chain) mediated modification of protein is involved in regulating target cell autophagy, apoptosis, cellular senescence, pyroptosis, necroptosis, and ferroptosis, and it is brought into sharp focus for its significant impact on different diseases. This paper will detail the linear ubiquitination modified protein substrate process, reveal its regulatory role in multiple cell fates, and define it as a potential therapeutic target for different diseases.

## Composition structure of LUBAC

LUBAC is the only ubiquitin E3 ligase complex to date that has been found to function on linear ubiquitin chain. It is composed of three parts: the heme-oxidized IRP2 ubiquitin ligase 1 L (HOIL-1L, approximately 123 kDa, 91.2% amino acid sequence conservation between humans and mice) encoded by RanBP-type and C3HC4-type zinc finger containing 1 (RBCK1), HOIL-1 interacting protein (HOIP, the molecular weight at 58 kDa, 86.5% conservation) encoded by Ring finger protein 31 (RNF31), and Shank-associated RH-domain interacting protein (SHARPIN, about 40 kDa, 73.6% conservation) encoded by SHARPIN [5, 17, 18]. The HOIP belongs to the RBR family due to the RBR domain. It interacts with E2-Ub through the RING1 domain and transfers Ub to catalytic Cys residue on the RING2 via Ub-thioester bond. Then, the donor Ub links to the acceptor Ub to form linear chain under the linear Ub determining domain (LDD) recognizing the acceptor Ub [19]. The HOIL-1L is also RBR E3 ligase, however it can downregulate LUBAC activity via monoubiquitination of all three subunits. The linear Ub chains mediated by HOIP prefer to binding to monoubiquitin, which could compromise the function of LUBAC [20]. Nonetheless, HOIL-1L and SHARPIN are both necessary to stabilize the complex [21]. The interaction between ubiquitin-associated domain (UBA) of the HOIP and ubiquitin-like domain (UBL) of the HOIL-1L and SHARPIN maintains the integrity of the LUBAC complex, and the HOIL-1L attaches to SHARPIN through the LUBAC-tethering motif (LTM) forming a single globular domain that stabilizes trimeric LUBAC. Besides, the UBL of the SHARPIN could bond to nuclear protein localization protein 4 zinc finger (NZF) [5]. The three components have their respective structural domains, which bind to each other for stabilizing LUBAC and exerting its function.

The functions of the LUBAC are known to be closely related to inflammatory conditions in humans. Mutation of the sharpin gene, is also called “cpdm mice”, spontaneously develop chronic proliferative dermatitis (CPDM) that manifested as skin inflammatory with epidermal hyperplasia, hyperkeratosis and the accumulation of dead keratinocytes. Comparatively, loss-of-function mutations in HOIP or HOIL-1L are rare but efficiently change LUBAC complex structure and cause a severe multiorgan autoinflammatory disease in humans.

## Composition of OTULIN and CYLD

Ubiquitination modification is a dynamically reversible process strictly regulated by deubiquitinating enzyme (DUB). Admittedly, linear ubiquitin chains on proteins can be removed by the DUBs OUT deubiquitinase with linear linkage specificity (OTULIN) and cylindromatosis gene (CYLD).

The OTULIN, encoded by FAM105B, comprises of ovarian tumor (OUT) domain and PUB interacting motif (PIM) domain. The OTU domain has three highly conserved residues (Cys129, His339, Asn341), which makes OTULIN active via bonding only M1 ubiquitin, and it explains the selective specificity linear chains catalysis of OTULIN. The OTULIN directly binds to the N-terminal PUB domain of HOIP via its PIM domain. This interaction is negatively regulated by Tyr56 phosphorylation of PIM domain [5]. Human patients with mutation of OTULIN function develop a systemic autoinflammatory pathology termed OTULIN-related autoinflammatory syndrome (ORAS) [22] represented as rashes, joint inflammation, as well as leukocytosis. The depletion of OTULIN can directly regulate the activation of LUBAC and effect related diseases progress. However, it is not yet clear whether this effect is positive or negative. Although M1-linked chain act as scaffolds for modifying proteins, the auto-ubiquitination in LUBAC can also happen what will impede the formation of M1-linked chain. Therefore, the catalyze of OTULIN on auto-ubiquitination LUBAC is positive to linear ubiquitination. Growing researches are being conducted to uncover the relationship between OTULIN and LUBAC.

CYLD belongs to Ub-specific protease (USP)-type DUB, and also negatively regulates linear ubiquitin. Different from direct interaction between OTULIN and HOIP, CYLD binding to PUB domain of HOIP requires spermatogenesis-associated 2 (SPATA2) for bridge factor [23]. Besides, CYLD can disassembles both M1-Ub and K63-Ub [24], even weak influences K11-Ub and K48-Ub [25], while OTULIN specifically removed M1-Ub chains. Given that OTULIN and CYLD share the same binding site in HOIP, they counteract each other for deubiquitylation of LUBAC. CYLD is critical to regulating a variety of signaling pathways that participate in inflammation, immunity and even cancer. Especially, the mutation of CYLD gene causes a broader spectrum of skin tumour syndromes was named CYLD cutaneous syndrome (CCS).

**Fig. 2:**
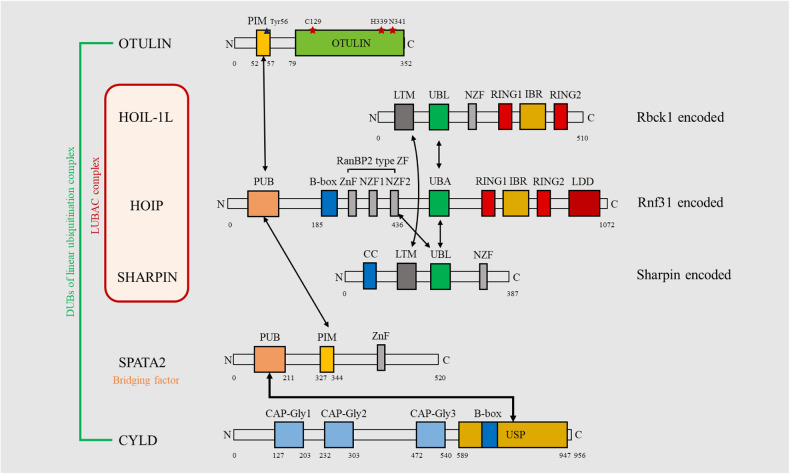
Domain organization of LUBAC subunits and associated DUBs. As the main functional unit of LUBAC, the HOIP contains 5 domains: C-terminal linear ubiquitin chain determining domain (LDD), RBR domain, ubiquitin-associated domain (UBA), RanBP2 type zinc finger (ZnF zinc finger, NZF nuclear protein localization protein 4 zinc finger), and PNGase/UBA or UBX-containing protein (PUB). HOIL-IL comprises ubiquitin-like domain (UBL), RBR domain, NZF, and LUBAC-tethering motif (LTM). SHARPIN is composed by UBL, NZF, LTM, and N-terminal coiled-coil domain. The OTULIN consists of an OTU domain and an N-terminal PUB-interacting motif (PIM) domain. Cys129, His339 and Asn341 are three highly conserved residues of OTU domain for active binding. The Tyr56 phosphorylation of PIM domain negatively regulates the interaction between OTULIN and LUBAC. The CYLD comprises of three cytoskeleton-associated protein glycine-rich domain (GAP-Gly) and N-terminal Ub-specific protease (USP) domain, and it triggers LUBAC-DUB interphase via a bridging factor named SPATA2. The SPATA2 contains PUB, PIM, and ZnF domains. The arrows indicate the interaction of domains.

It is worth noting that the deubiquitinase USP10 can interact with NEMO (NF-κB essential modulator, also called IKKγ) and lead to removal of NEMO-attached linear polyubiquitin chains [26]. The limited literatures can only demonstrate the deubiquitylation of USP10 on a specific substrate NEMO, and more studies are needed to investigate that USP10 may be a third deubiquitinating enzyme capable of participating in linear ubiquitination. The integrity of LUBAC and its deubiquitinases are illustrated in Fig. 2, and the small molecules and drugs that interfere with linear ubiquitination are listed in Table 1.

**Table 1:** The small molecules and drugs that interfere with linear ubiquitination.

Linear ubiquitination components		Linear ubiquitylation agonist	N/A
		Linear ubiquitylation antagonist	ABIN-1 [112], TIGAR [113]
LUBAC		Agonist	N/A
		Antagonist	Gliotoxin [105], HOIPIN-1/8 [59, 114], Aureothricin [63]
	HOIP	Agonist	Cisplatin [115]
		Antagonist	Thiolutin [67], Compound 5/11a [116]

Over the past decades, several forms of cell death such as apoptosis, necroptosis, autophagy, pyroptosis, and ferroptosis were identified. They have physiological and pathological functions, and their main characteristics and differences are presented in Table 2. The cellular lifespan can range from a few days to many years, which corresponds to the occurrence of various acute and chronic diseases. Influencing cell death via regulating critical proteins and signaling pathways has become an important way to explore potential treatment methods for relative diseases. As a post-transcriptional modification process, linear ubiquitination also plays a vital role in regulating cell death. Recent reports have shown that LUBAC mediated linear ubiquitination have involved in apoptosis, pyroptosis, necroptosis, ferroptosis, autophagy and cellular senescence via targeting key proteins for proteasomal degradation or allowing them to function as a scaffold to recruit other protein and regulate downstream signaling [5, 27].

**Table 2:** Characteristics and differences between apoptosis, necroptosis, autophagy, pyroptosis, and ferroptosis.

Cell death	Morphological features	Biological features	Trigger inflammation	Major regulators
Apoptosis	The cytoplasm shrinks, chromatin condensation, nuclear fragmentation, membrane blebbing and the formation of apoptotic bodies.	Extrinsic pathways: TLRs, TNF, FasL activation; Intrinsic pathways: caspases cleaved and cytochrome c release	No	Caspase-2/3/6/7/8/9/10, BAX, BAK, Bcl-2, p53
Necroptosis	The cell swells, pore formation on membrane, nucleus ruptures, DNA release, membrane ruptures and the cell content release to induce inflammatory reactions.	The TLRs, TNF pathways activation, necrosomes assembly, and MLKL oligomerize.	Yes	RIPK1/3, MIKL
Autophagy	Formation of double membrane autophagosomes, then combine with lysosome to auto-phagolysosomes.	Autophagic flux elevates, lysosomal activity, and mitochondria damaged.	No	ATGs, LC3, p62, Pink1, Parkin2
Pyroptosis	The cell swells, nucleus shrinks, DNA breaks, pore formation on membrane, membrane ruptures and the cell content release to induce inflammatory reactions.	Inflammasomes formation, gasdermin cleavage, and IL-1β, IL-18 release.	Yes	Caspase-1/3/4/5/8/11, gasdermin, NLRP2/3, IL-1β, IL-18
Ferroptosis	The cell membrane breaks and bubbles, the mitochondria become smaller, the mitochondrial membrane density increases and cristae decreases.	Iron and ros accumulation, lipid peroxidation, and Xc-system/GSH/GPX4 pathway inhibition.	Yes	GPX4, Nrf2, SLC7A11, P52, FSP1, ACSL4

## Regulation of apoptosis by linear ubiquitination

Apoptosis is the best-described form of programmed cell death (PCD) owing to the activation of catabolic enzymes downstream of mitochondrial cytochrome c release, which leads to rapid destruction of cellular structures and organelles, membrane blebbing, and nuclear chromatin condensation or even nuclear fragmentation [28]. Mechanistically, apoptosis is triggered through two major pathways: the intrinsic and extrinsic pathways. Existing studies suggest that linear ubiquitination is more affecting the extrinsic pathways. The extrinsic pathway is regulated by membrane receptors included Toll-like receptors (TLRs), tumor necrosis factor (TNF) receptor 1 (TNFR1), death receptor. Upon coupling with their ligands, the receptors oligomerize via TNFR1-associated death domain (TRADD) and receptor-interacting serine/threonine-protein kinase 1 (RIPK1) to form platforms at the cell surface [29]. Then TRADD facilitates recruitment of TRAF2, which provides a binding and activation platform for cellular IAP1/2(cIAP1/2). The cIAP1/2 as a E3 enzyme ubiquitinates RIPK1 and themselves for K63-polyubiquitin chain [30], which serves as a binding platform for linear ubiquitin chain assembly complex LUBAC, TAK1/TABs, and NEMO/IKK recruitment. LUBAC binds to NEMO and conjugates linear polyubiquitin chains onto specific Lys residues in the CC2-LZ domain in a Ubc13-independent manner [31]. Thereby LUBAC can ubiquitinate the NEMO and facilitate the recruitment of additional NEMO/IKK complexes. The IKK complex can be phosphorylated by TAK1 and then phosphorylates its downstream IκB, allows the proteasomal degradation mediated by K48 polyubiquitin chain, which releases the NF-κB and translocate it into nucleus for transcription of pro-inflammatory and anti-apoptosis gene [32, 33]. At early stages of recruitment of TRADD, ubiquitination of RIPK1, and release of Caspase-8, the regulatory protein FLIP ubiquitinated by LUBAC can limit the proapoptotic function via promoting NF-κB dependent signaling pathway [34, 35]. In addition to TNFR1/2 [36], LUBAC also regulates the CD40, TLRs, and interferons to induce canonical NF-κB signaling pathway [37]. Besides, LUBAC mediated NOD1/2 signaling pathway can also induce the MAPK and NF-κB activation and control the cell apoptosis. In response to NOD1/2 activation by peptidoglycans (PGN), X-linked inhibitor of apoptosis protein (XIAP) and cIAP1/2 were recruited to the receptor to RIPK2. XIAP and cIAP1/2 ubiquitylates RIPK2 for K63 polyubiquitin chain, and facilitates the LUBAC recruitment and activation of MAPK and NF-κB signaling pathway [38]. Above all, they have same characteristic that linear ubiquitination of NEMO/IKK complexes and activation of the NF-κB signaling pathway is the critical for LUBAC regulating cell apoptosis.

**Fig. 3:**
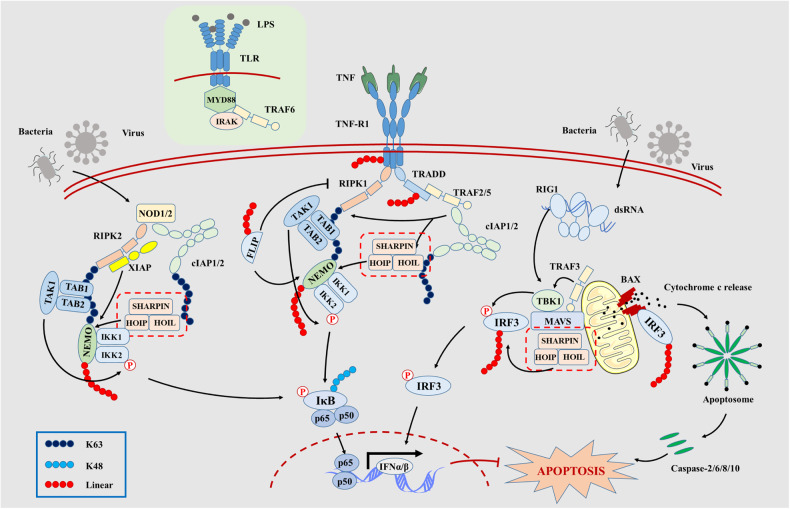
Apoptosis is triggered by intrinsic and extrinsic pathways with linear ubiquitination. When TNF-α binding to TNFR (or TLR activated by LPS), the TRADD, TRAF2/5 are recruited for signaling complex platform to activate cellular IAP1/2. The cIAP1/2 ubiquitinates RIPK1 and themselves for K63-polyubiquitin chain which anchors LUBAC for M1 linked polyubiquitin chains. Thereby LUBAC can ubiquitinate the NEMO and promote K48 mediated degradation of IκB. The free p65/p50 translocate into nucleus andfacilitate downstream NF-κB activation. In the cytoplasm, NOD1/2 activation leads to the recruitment of RIPK2, XIAP, and cIAP1/2, and cIAP1/2 ubiquitylates RIPK2 for LUBAC catalyzing NF-κB activation as like TNFR- NF-κB signaling pathway. RNA viruses can trigger the RIG-I-like receptor (RLR) pathway, which activates the transcription factor, IRF3. The recruitment of RIG-I to MAVS leads to activation of cytoplasmic kinase TBK1. The activated TBK1 phosphorylates IRF3 to translocate from cytoplasm to the nucleus and induces IFN-stimulated response. Besides, IRF3 recruited by the LUBAC complex alongs with MAVS, TRAF3, and TBK1 can trigger RIG-I induced pathway of apoptosis (RIPA), in which ubiquitinated IRF3 binds to Bax and translocates it into mitochondria causing the release of cytochrome c.

The intrinsic pathway is triggered by the release of cytochrome c into cytosol owing to the pore formation on the mitochondrial outer membrane in the control of the oligomerization of BAK/BAX. The cytochrome c, Aparf-1, and procaspase-9 are assembled to apoptosome, which activates a variety of caspases (caspase-2/6/8/10) and triggers cell apoptosis [29]. Current research reports that linear ubiquitinated IRF3 could bind to Bax and translocate it into mitochondria causing the release of cytochrome c [39]. The regulation of linear ubiquitination on cell apoptosis is depicted as Fig. 3. Commonly, RLR signaling activation could induce IFN-stimulated response including enhancing NF-κB activation [40, 41].

A variety of researches have investigated that LUBAC mediated linear ubiquitination participates in the regulation of cell apoptosis in different ways due to the complex structures of LUBAC. Genetic loss of LUBAC members or its DUBs can affect inflammatory phenotypes and cell fate. Majority of current researches demonstrate that linear ubiquitination is crucial for cell apoptosis via regulating nuclear factor NF-κB pathway. Different teams have verified that linear ubiquitination plays an important role in different diseases by intervening in the formation of the LUBAC complex or in regulating the activation of its exclusive deubiquitinases. After determining the vital role of linear ubiquitin chain assembly complex in TNF-R1 signaling pathway [32, 33] and downstream NEMO mediated NF-κB activation [31, 42, 43], SHARPIN, component of LUBAC, was found that negatively associates with TRAF2-mediated NF-κB activation [44–47]. It initially explained that the formation pattern of chronic proliferative dermatitis (cpdm). Another research has shown that sharpin can regulate keratinocytes apoptosis through affecting the recruitment of TRADD/FADD and activation of caspase-8 [48]. RNF31 regulates skin homeostasis by protecting epidermal keratinocytes from cell death [49]. Similarly, TNFα induced activation of NF-κB was reduced in hepatocytes from sharpin-deficient mice, which led to strongly exacerbated liver damage [50]. The impaired LUBAC formation may be one of the molecular mechanisms underlying the enhanced apoptotic response of hepatocyte in methionine and choline deficient (MCD) diet-induced NASH livers [51]. Overexpression of SHARPIN in prostate cancer cells promoted cell growth and reduced apoptosis through NF-κB/ERK/Akt pathway and apoptosis-associated proteins [52] and CCl_4_- or acetaminophen-induced liver cirrhosis [53]. The linear ubiquitin chain assembly complex serves as a previously undescribed tumor suppressor in the liver, restraining TNFR1-independent apoptosis in hepatocytes [54]. OTULIN prevents liver inflammation and hepatocellular carcinoma by inhibiting FADD- and RIPK1 kinase-mediated apoptosis in hepatocytes [55]. Besides, the TNFR signaling mediated cell apoptosis can also be regulated by intervening the HOIP gene expression [56]. Under cellular stress, the parkin, a RING-between-RING-type E3 ubiquitin ligase, is recruited to the linear ubiquitin assembly complex and increases linear ubiquitination of NEMO, and regulates the antiapoptotic pathway [57]. Another research reported that the CCCH-type zinc finger-containing protein MCPIP1 (monocyte chemotactic protein-1-induced protein-1; also known as ZC3H12A) upregulation reduces NEMO linear ubiquitylation via recruiting USP10 to remove NEMO-attached linear polyubiquitin chains, resulting in decreased activation of IKK and NF-κB [26]. For neurodegenerative diseases, optineurin (OPTN) regulates both NF-κB activation and apoptosis via linear ubiquitin binding, and the loss of this ability may lead to amyotrophic lateral sclerosis (ALS) [58]. Genetic ablation of HOIP or treatment with a LUBAC inhibitor, HOIPIN-8, suppressed the cytoplasmic aggregation of TDP-43, which eliminates TNFα mediated NF-κB activity and ameliorates TDP-43 induced ALS [59]. T cell-mediated adaptive immune responses are also effected by LUBAC ligase activity, survival of mature T cells depends on signaling through HOIP [60] and linear ubiquitin chain assembly complex coordinates late thymic T-cell differentiation and regulatory T-cell homeostasis [61]. Subsequent experiment demonstrated that linear ubiquitin chain assembly complex can regulate the B cell responses and B1b cell development [62, 63]. LUBAC controls the cFLIP expression and inhibits the function of caspase 8 and IL-21-activated caspase 9, thereby suppressing apoptosis of CD40 and IL-21-activated B cells and promoting GC B cell survival [64]. OTULIN-depleted cells spontaneously accumulated M1-Ub on LUBAC components, and NOD2 or TNFR1 stimulation led to extensive M1-Ub accumulation on receptor complex components [65]. LUBAC inhibited the expression of NR6A1 by promoting its linear ubiquitination, thereby dephosphorylating RIPK3 and consequently inhibiting the vascular smooth muscle cell (VSMC) apoptosis [66]. Genetic deletion and pharmacologic inhibition of E3 ubiquitin ligase HOIP impairs the propagation of myeloid leukemia [67]. Above all, the linear ubiquitination mediated cell apoptosis mostly is regulated by NEMO/NF-κB signaling pathway or its related regulation factors which comes to TNFR, TLR, and NOD signaling pathways. A few researches investigate that FADD/TRADD induced caspase-8 and RIG signaling mediated cytochrome c releasing from mitochondria, independent of NEMO/NF-κB signaling pathway, are also involved in the linear ubiquitination mediated cell apoptosis. Influencing apoptosis by regulating the linear ubiquitination may be a potentially effective way to treat diseases such as dermatitis, liver disease, and inflammation.

## Regulation of necroptosis by linear ubiquitination

**Fig. 4:**
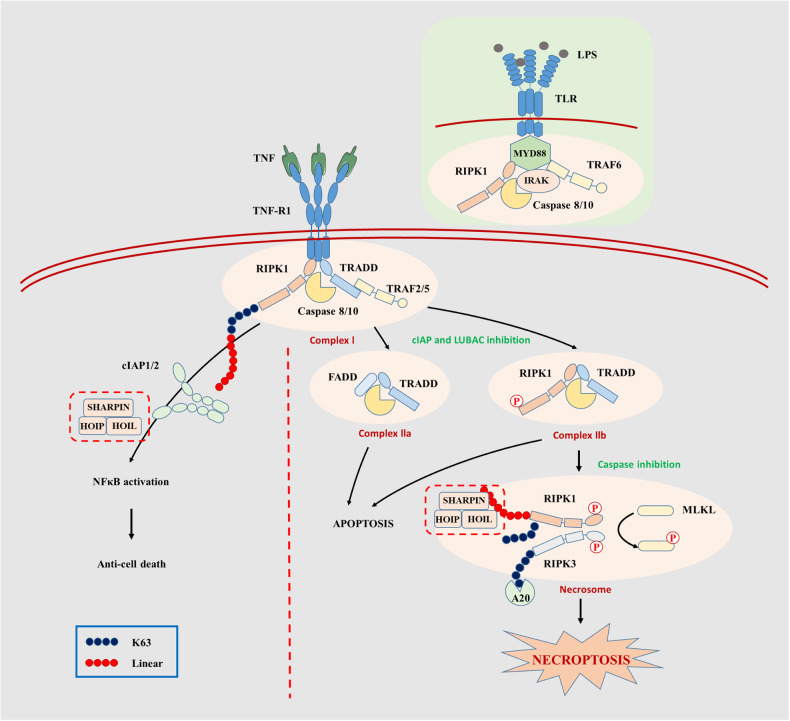
Necroptosis is triggered in the control of LUBAC activation at critical stages. Upon TNFRs or TLRs activation, the complex l usually involves in LUBAC mediated NEMO polyubiquitination and downstream NF-κB activation for apoptotic inhibition. Nevertheless, if the cIAP and LUBAC lose their function, the RIPK1 will escape from complex l and form a secondary cytoplasm complex called complex ll. Complex ll is divided into lla or llb distinguished by RIPK1. To this situation, the caspase 8/10 still can induce apoptosis. While caspases activation is suppressed, RIPK1 engages RIPK3 to form the necrosome that initiates necroptosis process. Meanwhile RIPK1 also contains M1 polyubiquitin chain in necrosome.

Necroptosis is a novel form of programmed cell death that is triggered when apoptosis is habited [68]. Similar to apoptosis, necroptosis can be induced by TNFα, interferons, TLRs or microbial infection [29]. Upon TNFα binding to TNFR1, the TRADD, TRAF2/5, and cIAP are recruited to form the receptor-associated signaling complex-l. Within complex l, the E3 ligase cIAP can promote ubiquitination of RIPK1 with K63 polyubiquitin chains. RIPK1 ubiquitination act as platforms to allow the binding of LUBAC that catalyze the activation of the NF-κB signaling pathway for anti-apoptosis. However, if cIAP lose its function on RIPK1 ubiquitination, RIPK1 will escape from complex l and form a secondary cytoplasm complex called complex ll which contains RIPK1, TRADD, FADD, Caspase 8/10, and RIPK3 [69]. Complex ll is divided into two categories, one is complex lla, and the other is complex llb according to whether it depends on RIPK1 or not [70]. The caspase 8/10 still can promote apoptosis so far, and caspase-8 is crucial for cleaving RIPK1 to limit apoptosis and necroptosis [71]. Upon caspase activation is inhibited, RIPK1 engages RIPK3 to form the necrosome. Following autophosphorylation of RIPK1 and RIPK3, the MLKL were recruited and phosphorylated. Phosphorylated MLKL undergo oligomerization and translocate to plasma membrane where it induces membrane rupture and cell necroptosis. As shown in Fig. 4, necroptosis is triggered in the control of LUBAC activation at critical stages.

Translocation of RIPK1 to the cytoplasm and association of RIP1 with the necrosome is believed to correlate with deubiquitylation of RIPK1. The importance of cIAP- and LUBAC-mediated ubiquitination in preventing uncontrolled RIPK1 activation and limiting necroptosis has been validated in different cell/ mouse models. TRA/GO induces oxidative stress and strong HER2 signaling to elicit immediate degradation of both cIAP and caspase 8, leading to activation of necroptosis [72]. HOIP deficient (HOIP-KO) mouse embryonic fibroblasts (MEFs) can develop TRAIL-mediated apoptosis and necroptosis [73]. The lethal dermatitis caused by keratinocyte-specific HOIL-1 is mediated by RIPK1 kinase dependent apoptosis and necroptosis [74]. Deficiency in SPATA2, a constitutive direct binding partner of HOIP that bridges the interaction between CYLD and HOIP, augments transcriptional activation of NF-κB and inhibits TNF-α-induced necroptosis [75, 76], because of promoting M1 ubiquitination of RIPK1 [77]. OTULIN promotes rather than counteracts LUBAC activity by preventing its auto-ubiquitination with linear polyubiquitin [78], and OTULIN inhibits RIPK1-mediated keratinocyte necroptosis to prevent skin inflammation in mice [79].

Otherwise, Almagro et al. found that RIPK1 also can be ubiquitinated with K63 and linear polyubiquitin chains in necrosome during necroptotic signaling [80]. It is interesting to figure out the difference between poly-ubiquitination of RIPK1 in complex l and necrosome. Chan al et has showed that CYLD-deficiency also affects ubiquitination of RIPK1 in necrosome, which demonstrates the role of CYLD in hydrolyzing M1- and K63-linked Ub chains in both complex l and necrosome [81]. Indeed, cIAP1 regulates RIPK1 recruitment to the necrosome without directly affecting RIPK1 ubiquitination, however HOIP and HOIL1 partially mediate linear ubiquitination of RIPK1 in the necrosome, while not essentially to cell necroptosis [80]. Therefore, we speculate that the linear ubiquitination of RIPK1 adverse to cell necroptosis via decreasing activation of RIPK1 in complex l. Remarkedly, RIPK1 has been reported to contain both M1- and K63-linked poly Ub chains [81], and necrosome associated RIPK3 is conjugated with K63-linked Ub chains [82]. A20, removes K63 poly Ub chains, can inhibit the formation of RIPK1-RIPK3 complexes to prevent necroptosis. It seems that polyubiquitination of RIPK1 and RIPK3 in necrosome have opposite effect on necroptosis induction [70], so we still believe the function of polyubiquitination of RIPK1 in complex l and necrosome is indefinite.

## Regulation of autophagy by linear ubiquitination

**Fig. 5:**
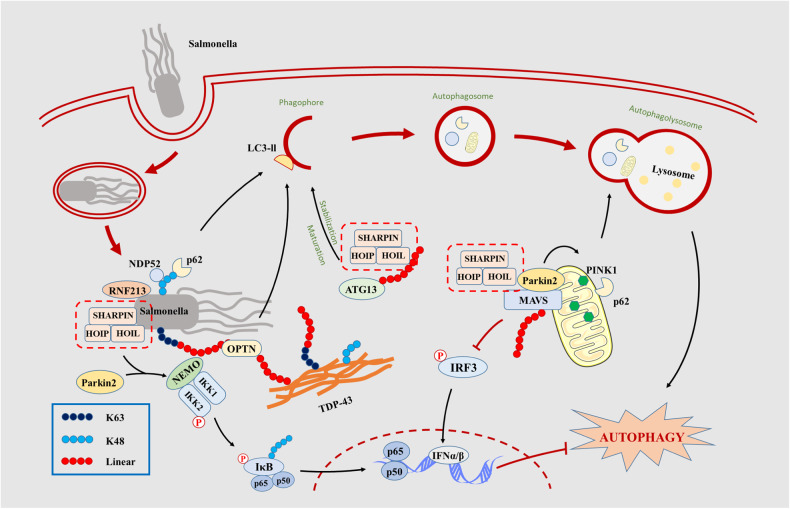
Autophagy is triggered by several LUBAC mediated signaling pathways in response to bacteria invasion. After salmonella invades into cytosol via SCVs package, LUBAC is recruited to salmonella through recognizing ubiquitin chains catalyzed by RNF213, and then M1 linked polyubiquitin chains extend on the pre-existing ubiquitin. The existing M1 polyubiquitin chains activates NF-kB signaling activation for apoptotic inhibition. Besides, optineurin (OPTN) can recognize linear polyubiquitin chains on salmonella and TDP-43 to bind to LC3, and push forward autophagosome-lysosome degradation process. Meanwhile LUBAC and OTULIN cooperatively regulate autophagy initiation and autophagosome maturation by mediating the linear ubiquitination and the stabilization of ATG13. Parkin is commonly recruited to mitochondria to induce mitophagy, and it is recruited to mitochondria and induces LUBAC to catalyze linear ubiquitin chains on mitochondrial antiviral signaling protein (MAVS), which abolishes IRF3 phosphorylation and regulate IFNα/β mediated cell autophagy.

Autophagy is a self-eating system that is essential for the removal of protein aggregation, intracellular bacteria, and damaged cellular organelles [83, 84]. Autophagy is characterized by the formation of double membrane vesicles called autophagosome, which engulf the cytoplasmic contents to lysosomes for destruction. When surrounding bacteria invading cells, autophagy receptors associated with ubiquitin-coated bacteria and target them to the autophagosome membrane, allowing the following lysosomal degradation (Xenophagy) [85]. Meanwhile, upon microbial infection or stress, the impaired mitochondria can also be cleared via autophagosome called mitophagy [86]. When salmonella invades cells, the bacteria reside in salmonella-containing vacuoles (SCVs), then the SCVs rupture and the salmonella are exposed to cytosol. RNF213 directly conjugates ubiquitin to cytosolic salmonella [87]. LUBAC is recruited to salmonella by recognizing ubiquitin chains catalyzed by RNF213 on the bacteria, and then conjugates M1 linked polyubiquitin chains to the pre-existing ubiquitin [88, 89]. These M1 polyubiquitin chains recruit NEMO for subsequent NF-κB signaling activation [90, 91] and optineurin (OPTN) for linking to LC3 [92] in order to restrict bacterial proliferation via autophagosome-lysosome degradation. As another critical factor regulating autophagy, OPTN can also recognize the linear polyubiquitin chains on TDP-43 skein-like inclusions and promote the autophagosome mediated protein degradation [59, 93]. Besides, recruitment of the autophagy cargo receptors NDP52 and p62 can directly facilitate the formation of autophagosome. NDP52 can bind to galectin 8 [94], and connect glycans exposed on damaged host membranes, which recognize bacteria and bridge it to LC3 in autophagosome. Parkin, an RBR E3 ligase, is commonly recruited to mitochondria to induce mitophagy [95], and it recently has been demonstrated that is recruited to the LUBAC and increases linear ubiquitination of NEMO, then facilitates the NF-κB mediated cellular stress defense [57]. As a result, NF-κB regulates transcription of OPA1, which controls the fission and fusion of mitochondria and maintains mitochondrial integrity [96]. As mentioned above that RLR signaling activation dependent on mitochondria could induce IFN-stimulated response. During the process, parkin is recruited to mitochondria and induces LUBAC to catalyze linear ubiquitin chains on mitochondrial antiviral signaling protein (MAVS), which abolishes IRF3 phosphorylation [97]. Current research has reported that LUBAC and OTULIN cooperatively regulate autophagy initiation and autophagosome maturation by mediating the linear ubiquitination and the stabilization of ATG13 [98], what verified that linear ubiquitination is able to directly regulate autophagy. Above all, linear polyubiquitin chain mediated autophagy comes to a variety of modes such as recruiting autophagic receptors (OPTN, NDP52, and p62), directly ubiquitinating ATG13 to affect its maturation and stabilization, and regulating mitochondria function. The concrete regulatory relationships between autophagy and linear ubiquitination are presented in Fig. 5. A few diseases including virus or bacterial infection [90, 91, 97] and amyotrophic lateral sclerosis [58, 93] associated with linear polyubiquitin chain mediated autophagy are currently studied, and they provide novel perspectives for the treatment of neurodegenerative diseases and virus or bacterial disorders.

## Regulation of pyroptosis by linear ubiquitination

Pyroptosis is a novel form of PCD, which is characterized by DNA fragmentation, chromatin condensation, cell swelling, and leakage of cell contents [99]. It is an inflammatory form of cell death triggered by intracellular sensors such as NLRP3 inflammasome, caspase-4/5/11, or others [29]. In the canonical pathway, danger-associated molecular patterns (DAMPs) and pathogen-associated molecular patterns (PAMPs) stimulate cells and arouse intracellular signaling, which make various inflammasomes activate the caspase-1. The inflammasome is a polyprotein complex containing a sensor protein (such as NLRP1, NLRP3, NLRC4, AIM2 and Pyrin), an adapt protein named apoptosis-associated speck-like protein containing CARD (ASC), and pro-caspase-1. ASC is a double adaptor protein which contains caspase recruitment domain (CARD) and pyrin domain (PYD). The sensor protein containing PYD domain and pro-caspase-1 containing CARD domain are assembled by ASC through the interaction of CARD-CARD and PYD-PYD. Then, the active caspase-1 cleaves pro-IL-1β, pro-IL-18, and GSDMD for mature IL-1β and IL-18, as well as N-GSDMD that self-assemble into membrane pores. Finally, mature IL-1β and IL-18 flow out of the GSDMD pores leading to inflammatory reaction or cell death. In the caspase-4/5/11-dependent noncanonical inflammasome pathway, the exogenous LPS directly activates caspase-4/5/11, and the active caspases similarly cleave the GSDMD and trigger pyroptosis.

Linear ubiquitination was reported to prevent inflammation and regulate immune signaling, in which the NOD2 signaling would participate [100, 101].Recent study has demonstrated a novel HOIL-1L function as an essential regulator of the assembly of the NLRP3/ASC inflammasome in primary bone marrow derived macrophages (BMDMs). Importantly, ASC is identified as a novel LUBAC substrate. The loss of functions in HOIL-1L^−^^/−^ mice results in resistance to peritonitis and contributes to survival upon LPS-induced systemic inflammation [102]. Another study shows that SHARPIN, a member of LUBAC complex, controls NLRP inflammasome activation and downstream caspase-1, caspase-11 (human caspase-4), and IL-1β by regulating its transcriptional priming, which could control NLRP3-associated inflammatory disorders [46, 103, 104]. The nuclear factor NF-κB transcription factors, leading to the expression of inflammatory factors, play a critical role in regulating their transcriptional priming [105]. Besides, activation of AMPK pathway via linear ubiquitination of LKB1 may be a novel target for NLRP3 inflammasome response [106]. Further research demonstrated that SHARPIN could bind caspase-1 and disrupt p20/p10 dimer formation, the last step of caspase-1 activation processing, thereby inhibiting maturation of IL-1β/IL18 in circulating mononuclear cells [107]. Another research has shown that HOIP forms a constitutive interaction with caspase-1 and mediates the linear ubiquitination of its CARD pro-domain [108]. Coincidentally, the three components of LUBAC (HOIP, HOIL-1L, and SHARPIN) all can target to critical proteins of pyroptosis.

## Regulation of ferroptosis by linear ubiquitination

Ferroptosis as an iron-dependent form of non-apoptotic cell death can occur through two major pathways, the extrinsic or transporter-dependent pathway and the intrinsic or enzyme-regulated pathway, which is caused by lethal lipid peroxidation under imbalance of oxidants and antioxidants [109]. Current report demonstrates that LUBAC deficiency sensitizes to ferroptosis by promoting GPx4 degradation and downstream lipid peroxidation. LUBAC binds and stabilizes GPx4 by modulating its linear ubiquitination both in normal condition and under oxidative stress [110]. The SPATA2/CYLD pathway has been identified that involving the deubiquitination of NCOA4 and enhancing ferritinophagy to promote doxorubicin-induced cardiomyocyte ferroptosis [111]. As mentioned above, ATG13 is a key substrate of LUBAC/OUTLIN and it is also possible to participate in cell ferroptosis [98]. Above all, linear ubiquitination of critical factors could regulate cell ferroptosis, which provides a potential treatment option for treating related diseases.

## Discussion

Linear ubiquitination is one of post-translational modifications recognized by M1 linked polyubiquitin chains that regulates multiple cellular processes. Different from other polyubiquitinations, the linear ubiquitin chain is generated by the sole E3 ubiquitin ligase LUBAC. Fortunately, the components of LUBAC have been studied preliminarily. We can monitor the related proteins through regulating the assembly of LUBAC or effecting its deubiquitinases (OTULIN and CYLD). Recent studies suggested that M1-linked chain act as scaffolds for modifying proteins, which explains the activation of signaling pathway under linear ubiquitination on critical proteins. Referring to previous literatures, the TNF-α mediated NF-κB signaling as an important target pathway of LUBAC regulates cell programmed death such as apoptosis or necroptosis. However, the growing evidence also investigates that linear ubiquitination is likely to control intercellular signaling and regulate other functions beyond the NF-κB activation. We have detailed the different regulation pathways other than NF-κB induced by linear ubiquitination in apoptosis, necroptosis, autophagy, pyroptosis, and ferroptosis. It also explains the fact that linear ubiquitination mediated diseases exceed the inflammatory and autoimmunity diseases but also involved in other tissues homeostasis. However, existing gene-targeting studies using cell-specific knockout mice can only demonstrate the relationship between linear ubiquitin modification and diseases, the concrete regulatory mechanism is still uncover owing to the molecular specificity and complexity of linear ubiquitination. Based on discussions triggered by multiple articles, three questions about the mechanism of linear ubiquitination regulating diseases are concluded following: (1) how substrates are recognized by LUBAC, (2) the role of polyubiquitin chains in effecting the function of substrates, (3) how to distinguish auto-ubiquitination in LUBAC during linear ubiquitination on substrates. In sight of multiple modalities of cell death occur in diseases as show in Table 3, in-depth exploration of linear ubiquitination is likely to provide novel therapeutic insights that might relieve patients pain with such diseases.

**Table 3:** Regulation of linear ubiquitination on genotypes and phenotypes in several modality of cell death.

Cell death	Genotype	Cell targeted	Substrate	Disease	Ref
Apoptosis	Sharpin	B cells, macrophages and mouse embryonic fibroblasts (MEFs)	NEMO/ NF-κB	Chronic proliferative dermatitis (cpdm)	[44–47]
	Sharpin^cpdm/cpdm^	Keratinocytes	TRADD/ FADD/caspase-8		[48]
	RNF31	Keratinocytes	TNFR1/ NF-κB	Skin inflammation	[49]
	Sharpin	Hepatocytes	NF-κB	Liver damage	[50]
	Sharpin	Hepatocytes	NEMO/ NF-κB	Nonalcoholic steatohepatitis (NASH)	[51]
	Sharpin	Hepatocytes	NEMO/ NF-κB	Liver cirrhosis	[53]
	Hoip	Hepatocytes	TNFR1	Hepatitis and hepatoma	[54]
	Otulin^LPC-KO^	Hepatocytes	RIPK1/ FADD/caspase-8	Liver inflammation, fibrosis, and liver cancer	[55]
	Sharpin	Prostate cancer cells	NF-kB/ ERK/ Akt	Prostate cancer	[52]
	HOIP^−/−^	endothelial cells	TNFR signaling	Vascularization and embryonic lethality	[56]
	Parkin	Mouse embryonic fibroblasts (MEFs)	NEMO/ NF-κB	Cell stress death	[57]
	MCPIP1	HEK293T	NEMO/ NF-κB		[26]
	Optineurin (OPTN)	HEK293T	NEMO/ NF-κB	Amyotrophic lateral sclerosis (ALS)	[58]
	HOIP deletion	Neuro2a cells	TDP-43/ NF-κB		[59]
	RNF31^Δlinear/Δlinear^	CD4^+^ T cells	NF-κB	T cell immune responses	[60]
	Optineurin (OPTN)	HEK293T and HeLa cells	NEMO/ NF-κB		[61]
	HOIP^FL^ mice	B cells	TLR4/ NF-κB	B cell immune responses	[62, 63]
	Sharpin^+/+^ and Sharpin^cpdm^	B cells	cFLIP/ Caspase-8/ IL-21		[64]
	Otulin	HEK293T	RIPK2/ NF-κB	Inflammatory	[65]
	LUBAC complex	Vascular smooth muscle cells (VSMCs)	NR6A1	Atherosclerosis	[66]
	HOIP deletion	Leukemia stem cells (LSCs)	NF-κB	Myeloid leukemia	[67]
Necroptosis	HOIP KO	Mouse embryonic fibroblasts (MEFs)	RIPK1	N/A	[73]
	SPATA2	BMDMs	CYLD/ HOIP/ TNF signaling		[75]
	SPATA2	A549, HEK293, HCT116, AND L929 cells	CYLD/ TNF signaling		[76]
	SPATA2^+/+^, SPATA2^−/−^	Primary MEFs and BMDMS	CYLD/ TNF signaling		[77]
	Hoip-1^E-KO^, Hoil-1^E-KO^	Keratinocytes	RIPK1	Skin inflammation	[74]
	OTULIN^E-KO^	Keratinocytes	RIPK1		[79]
Autophagy	RNF213/ LUBAC	HeLa cells	Parkin/ Xenophagy	Salmonella infection	[87]
	RNF213/ LUBAC	A549 and HFF-1 cells	IFN	Toxoplasma infection	[89]
	TDP-43/ LUBAC	Clinical samples	Optineurin (OPTN)	Amyotrophic lateral sclerosis (ALS)	[58, 93]
	Parkin/ LUBAC	HepG2, Huh7 and HEK293 cells	IRF3/ IFN signaling	Hepatitis B virus infection	[97]
	OTULIN	HEK293 FT and HeLa cells	ATG13	N/A	[98]
Pyroptosis	HOIL^−/−^	BMDMs	ASC	Peritonitis and system inflammation	[102]
	Sharpin^cpdm^	BMDMs	NLRP3 transcriptional priming	Dermatitis	[103]
	Sharpin^cpdm^ Nlrp3^−/−^ lce^−/−^(Caspase-1/11^−/−^)	Full body	Caspase-1/11 activation		[46]
	Sharpin^cpdm^ Il1b^−/−^	Full body	IL-1β activation		[104]
	shHOIP	Keratinocytes	CARD of caspase-1		[108]
	GST-SHARPIN	THP-1 cells	Caspase-1	Sepsis	[107]
	LUBAC inhibitor	THP-1 cells	NF-κB activation	Tuberculosis	[105]
	oe-HOIP	Chondrocytes	LKB1/AMPK	Osteoarthritis	[106]
Ferroptosis	LUBAC deficiency	MEFs	GPx4	N/A	[110]
	SPATA2/CYLD	Cardiomyocyte	NCOA4	Cardiotoxicity	[111]
